# Targeting H3K27me3 demethylase to inhibit Shh signaling and cholesterol metabolism in medulloblastoma growth

**DOI:** 10.3389/fonc.2022.1057147

**Published:** 2022-12-01

**Authors:** Hongshi Deng, Xueli Guo, Na Feng, Yi Luo, Bei Liu, Shuzhen Liu, Jiang I. Wu, Xuanming Shi

**Affiliations:** ^1^ School of Basic Medical Sciences, Anhui Medical University, Hefei, Anhui, China; ^2^ Department of Physiology, University of Texas Southwestern Medical Center, Dallas, TX, United States

## Abstract

Previously we uncovered the epigenetic regulation of medulloblastoma that low levels of H3K27me3 are required for Shh target gene expression and medulloblastoma growth. Since Jmjd3, an H3K27me3 demethylase, is responsible for maintaining low H3K27me3 at Shh target genes, targeting Jmjd3 could be an efficient way to inhibit Shh signaling and medulloblastoma growth. Here we show that the small molecule GSK-J4, an inhibitor of Jmjd3, significantly inhibited the expression of Shh target genes in Shh responsive cell models and primary cerebellar granule neuron precursors. GSK-J4 also significantly reduced the growth of primary Shh medulloblastoma cultures. Treating human medulloblastoma cell line DaoY by GSK-J4 led to cell cycle arrest at G0/G1 phase with decreased cells in S-phase. Tumor cell proliferation was significantly inhibited by GSK-J4 treatment. Gene expression analyses showed that GSK-J4 additionally constrained the expression of key genes in cholesterol biosynthesis. Our results highlight the possibility that targeting H3K27me3 demethylase Jmjd3 with GSK-J4 to inhibit Shh signaling and cholesterol metabolism is a potential application to treat Shh medulloblastoma.

## Introduction

Shh ligand covalently modified with N-terminal palmitoylation and C-terminal cholesterol plays essential roles in mammalian embryonic development, cell homeostasis and tumor formation (1). When Shh ligand binds to its receptor Patched (Ptch), Smoothened (Smo) is released from Ptch to activate transcription activator Gli1/2-Act. The translocated Gli1/2-Act in the nucleus activates Shh signaling by increasing the expression of general Shh target genes such as *Gli1*, *Ptch1* and *Hhip* (2, 3). During cerebellar development, Shh molecules are produced from Purkinje neurons and activate mitogenic target genes such as *N-myc* and *Ccnd1* in cerebellar granule neural precursors (CGNPs). Hence, Shh drives the proliferation of CGNPs and contributes to cerebellar development (4–6). During normal cerebellar development, Shh levels are properly modulated and reach a peak at an early postnatal stage. However, the overactive Shh signaling caused by mutations in Shh pathway genes such as *Smo* and *Ptch1* is the driving force for Shh-type medulloblastoma (7, 8). SmoM2 is a Smo mutant resulting in constitutively active Shh signaling (9). This mutant has been widely used to establish mouse Shh medulloblastoma tumor models (10, 11).

Medulloblastoma is the most common malignant pediatric brain tumor, and accounts for about 20% of all childhood brain tumors (12, 13). Genomic studies classified medulloblastoma into four subgroups: Wnt, Shh, Group 3 and Group 4 (8, 14–17). The Wnt and Shh groups were named according to the principally activated signaling pathways in the tumors. Wnt-type tumors arise from the embryonic brain stem and lower rhombic lip progenitor cells (18). In contrast, Shh-type tumors originate from CGNPs with active Shh signaling (19, 20). This type of tumor is often found in infants and adults and accounts for about 25% of all medulloblastoma (11). Group 3 tumor features large cell/anaplastic phenotype and is driven by oncogenes *c-myc* and *OTX2* (18, 21, 22). The cell origin of Group 4 medulloblastoma is thought to originate from unipolar brush cells (23–25). Current therapies for medulloblastoma consist of surgery, radiotherapy and chemotherapy. The standard radiotherapy includes craniospinal irradiation with a radiation boost to tumor bed. More often, radiotherapy medicated by Linac-based photons is being replaced by proton-beam based radiotherapy. Although prognosis is improved, patients still have severe long-term side effects (8, 26). Having a better understanding of both the active Shh signaling pathway and cell of origin provides possibilities of targeted therapies for medulloblastoma (27).

Epigenetic regulators play important roles in Shh signaling and medulloblastoma development (10, 11, 28–31). Previously we identified histone 3 lysine 27 trimethylation (H3K27me3) demethylase Jmjd3 as a key player in Shh target gene activation (10). We showed that Jmjd3 mediated H3K27me3 demethylation and epigenetic changes are required for the activation of Shh target genes in response to Shh stimulation (11, 31). Genetic knockout of Jmjd3 significantly inhibited Shh medulloblastoma growth in culture and in mouse models (10). Therefore, pharmacological inhibition of Jmjd3 could be a promising treatment option for medulloblastoma. Kruidenier et al. reported that an ethyl ester GSK-J4 inhibits H3K27me3 demethylase activities (32). By targeting Jmjd3, GSK-J4 has been successfully applied to inhibit the growth and proliferation of various tumors including pediatric brainstem glioma, chondrosarcomas, and lung adenocarcinoma in mouse xenograft models or *in vivo* studies (33–35).

In this report, we demonstrated that GSK-J4 efficiently inhibits Shh signaling and Shh-type medulloblastoma growth. We confirmed the notion in several Shh responsive cell models including immortalized fibroblasts NIH3T3, primary MEFs, normal CGNPs, primary mouse tumor cells, and human medulloblastoma cell line DaoY. In DaoY medulloblastoma cells, GSK-J4 additionally inhibits tumor cholesterol metabolism, which contributes to Shh signaling and cell proliferation. These results highlighted the potential translation to use the small molecule inhibitor GSK-J4 targeting H3K27me demethylase Jmjd3 for Shh-type medulloblastoma treatment.

## Materials and methods

### Mice

The *SmoM2* transgenic mice were purchased from Jackson Laboratory (Strain #:005130). *SmoM2 CAG-CreER* mice spontaneously develop Shh-type medulloblastoma (9).

### Cell lines, primary MEF, CGNP and medulloblastoma cell cultures

NIH3T3 cells, DaoY cells, wild type MEF cells, *Gli3^-/-^* and *Jmjd3^-/-^* MEF cells were cultured in DMEM containing 10% FBS, sodium pyruvate, penicillin, streptomycin, minimal amino acid, L-glutamine and 2-mercaptoethanol. *Jmjd3^-/-^* MEF cells were provided by Dr. T. Akira (36). Primary CGNP cultures were derived from dissociated P4 wild type mouse cerebella and cultured in DMEM/F12 media containing 25 mM KCl, N2, penicillin, streptomycin, and 10% FBS as previously described (31). For Shh induction, Shh conditioned media produced from Shh-CM 293T cells (37) were added to MEF and CGNP cultures at 1:20 dilution. NIH3T3 cells or primary MEF cells were treated with Shh in 0.5% FBS media for 24 hours before harvesting. Primary tumor cells were derived from dissociated SmoM2 medulloblastoma and cultured in DMEM/F12 media containing B27, N2, EGF, and FGF2. For GSK-J4 (XcessBio, M60063-2) treatment, media were mixed with indicated concentrations of the compound, and DMSO was used as a solvent control. The ATP assay for cell viability analysis was carried out as described (10).

### Western blot

Western blot was carried out as previously described (38). In detail, cultured cells were washed with ice-cold 1x PBS for three times, and lysed in RIPA buffer (50 mM Tris, pH 8.0, 150 mM NaCl, 0.05% SDS, 0.5% DOC, 1% NP-40). Cell lysates were separated on SDS-PAGE gels. Antibodies against Gli1 and Caspase3 were purchased from Cell Signaling and Protein Tech, respectively. HRP-conjugated secondary antibodies were from Jackson Immunology. GAPDH was detected as a loading control.

### Reverse-transcription quantitative PCR

RT-qPCR was performed as described (38). RNAs from cells or tissues were extracted with TRIZOL (Invitrogen). The concentration of RNA samples was determined with NanoDrop One (Thermo Scientific). cDNAs were synthesized by reverse transcription using Iscript. iTaq reagents were used for quantitative PCR. Both Iscript and iTaq were purchased from Bio-Rad. A Bio-Rad real-time PCR system (C1000 Thermal Cycler) was used for quantitative PCR. Levels of GAPDH mRNA were used to normalize input RNAs. Relative gene expression was calculated by ΔΔCt as previously described (38). Graphics shown are representative of experiments performed in triplicate. Sequences of PCR-primers are listed in extended data.

### Chromatin immunoprecipitation assay

ChIP experiments were performed as previously described (10). Dissociated cells were crosslinked with paraformaldehyde, and DNA was sonicated to fragments (200-1000 bp) in the nuclear lysis buffer using a Sonic Dismembrator 550 (Fisher Scientific) at level 3, output 10%, for 5 cycles of 7 sec on, 30 sec off, in ice water. An antibody against H3K27me3 (#39536, Active Motif) was used in the precipitation step. Precipitated DNA was captured using pre-blocked Protein G agarose beads (Pierce) in the presence of BSA and salmon sperm DNA, followed by purification and subjection to quantitative PCR. The primer sequences are CGCTCACTTCCCTCGTATATCCTTC, GGCAGTATAGGGTCCCTCAAGGG. 10% of input was used for positive control and normalizing results, which were presented as percentages of input.

### RNA-seq and bioinformatics analyses

RNA-seq analyses were performed in the Sequencing and Bioinformatics Center in Anhui Medical University. Total RNAs were extracted using TRIzol Reagents (Invitrogen), and mRNAs were separated using magnetic beads (Vazyme, N401). Reverse transcription was performed to obtain cDNA using SuperScript™ II (Invitrogen, # 18064014). cDNA libraries were established using the Tn5 DNA Library Prep Kit from Illumina (TRANS, KP101-11) and sequenced for PE150. Fastq data were analysed with Fastqc and processed using the pipeline described in the Linux system (39). The expression levels of genes were quantified with featureCounts (40), and the differentially expressed genes were called by DESeq2 program with a fold-change > 2 and padj <0.05. Data were analysed and presented using R studio for GO, KEGG, GSEA and STRING analyses.

### Immunofluorescence staining

DaoY cells were seeded on cover glasses at a density of 10^5^ cells/well in 24-well plates. Cells were treated with 10 μM of BrdU for 2 hours before staining. For staining, cells on cover glasses were fixed using methanol, then washed with PBS for 3 times. The cells were then treated with 2 M of HCl for 20 min, and blocked with 5% goat serum for 30 min at room temperature. The cells were incubated with mouse anti-BrdU antibodies (ABclonal, A1482) o/n at 4°C and then with GFP-Conjugated goat anti-mouse IgG antibodies (ZGGB-BIO, ZF-0312) for 30 min at 20°C.

### Cell cycle assay

The cells were cultured with GSK-J4 for 24 hours in 6 cm dishes at a density of 4 x 10^5^ cells per dish. Cell cycle assay was performed according to the manufacturer’s instructions (Beyotime, C1052). Stained cells were applied to a BD FACSVerse flow cytometer (BD Bioscience), and data were analysed using ModFit software.

### Statistical analysis

Each experiment was repeated at least three times except for RNA-seq analyses, data shown were from representative assays with 3 technical repeats. Data are expressed as means plus standard deviation (s.d.). Statistical analyses were performed using a two-tailed, unpaired Student’s *t*-test provided by Microsoft Office Excel. A *p* value of <0.05 will be considered significant.

## Results

### Shh signaling is impaired by GSK-J4 targeting H3K27me3 demethylase Jmjd3

**Figure 1:**
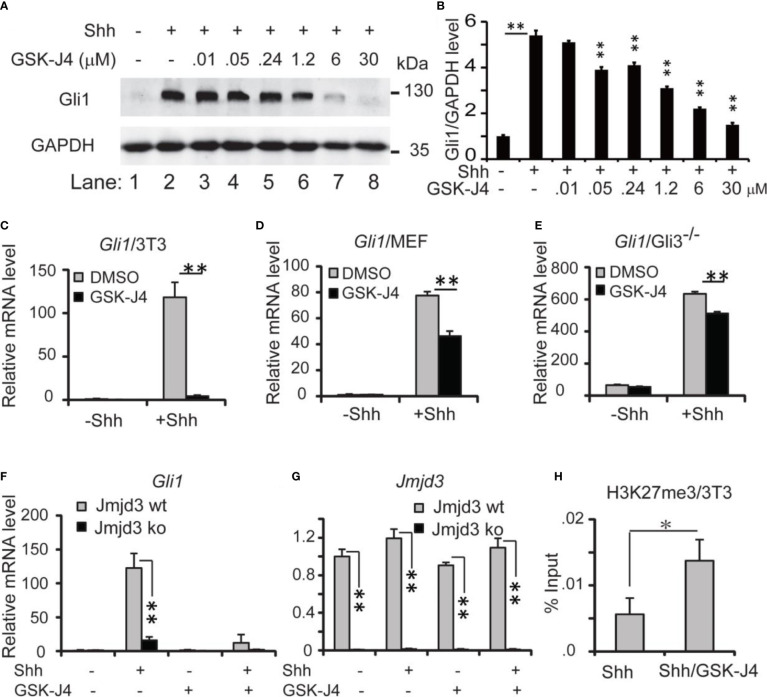
Shh signaling is impaired by GSK-J4 targeting H3K27me3 demethylase Jmjd3. **(A)** Effects of GSK-J4 at various concentrations on Gli1 protein levels. **(B)** Ratio of Gli1 protein levels against GAPDH in **(A)** calculated by Image J Significance is against Shh induced level (*rf* **Figure 1A**, lane 2) except for the one between **Figure 1A** lane 1 and **Figure 1A** lane 2. **(C)** GSK-J4 impairs Shh-induced *Gli1* mRNA expression in NIH3T3 cells by RT-qPCR analysis. **(D)** GSK-J4 inhibits Shh-induced *Gli1* mRNA expression in primary MEF cells. **(E)** GSK-J4 significantly inhibits Shh-induced *Gli1* mRNA expression in primary *Gli3^-/-^* MEF cells. **(F)** Shh-induced *Gli1* mRNA expression is impaired in primary *Jmjd3^-/-^* MEF cells, and GSK-J4 does not further decrease the *Gli1* mRNA level. **(G)** *Jmjd3* mRNA expression is reduced by *Jmjd3* knockout. **(H)** GSK-J4 raises the local H3K27me3 enrichment at mouse *Gli1* promoter region shown by ChIP q-PCR assay. The concentration of GSK-J4 is 30 μM if not indicated for all figures. Significance determined using Student’s *t*-test. **p < 0.01 and *p < 0.05.

Since Shh medulloblastoma is caused by over-active Shh signaling in cerebellum, modulating Shh signaling could be an effective way to constrain the tumor growth. Previously we reported that Jmjd3 plays an essential role in Shh signaling activation and Shh-medulloblastoma growth (10). Kruidenier et al. developed an H3K27me3 demethylase inhibitor, GSK-J4, to modulate Jmjd3 activity, making it possible to intervene the diseases that depend on Jmjd3 (32). To determine whether GSK-J4 can block Shh signaling by targeting Jmjd3, we chose the immortalized embryonic fibroblast NIH3T3 cells (41), which is responsive to Shh signaling and could activate Shh target genes such as *Gli1*, *Ptch1*, *Hhip*, and *Hck* (10, 11, 31, 38). Among them, *Gli1* encodes a transcription factor forming a positive feedback loop to further activate Shh/Gli1 target genes. The mRNA level of *Gli1* faithfully reflects the activities of Shh pathway (31), hence we use *Gli1* as a readout of the pathway. Previously 30 µM of GSK-J4 was applied to inhibit the proinflammatory macrophage response through targeting the jumonji domain of histone demethylase (32), we hence tested this concentration of GSK-J4 on NIH3T3 cells, and treated the cells with Shh conditioned media and various concentrations of GSK-J4 at 0.01, 0.05, 0.24, 1.2, 6, and 30 μM (**Figures 1A** **, B**). The Treatment of GSK-J4 (**Figure 1A**, lane 4-8) significantly reduced the Shh induced Gli1 protein levels (**Figure 1A**, lane 2) when the concentration of GSK-J4 is above 6 μM. The quantifications indicated that 30 μM of GSK-J4 is higher than the IC50 in NIH 3T3 cells for target gene Gli1 expression (**Figure 1B**).

Upon addition of Shh conditioned media to NIH3T3 cells, *Gli1* mRNA levels were significantly increased, indicating an activated Shh signaling pathway (**Figure 1C**). After cells were treated with 30 µM of GSK-J4 for 24 hours, *Gli1* expression was significantly decreased to ~10% (**Figure 1C**). Thus, GSK-J4 is a potential inhibitor of the Shh signaling pathway. Since the immortalized NIH3T3 cells were generated from mouse embryonic fibroblast (MEF), we carried out the GSK-J4 inhibition experiment using primary MEF cells. Similarly, the Shh-induced *Gli1* expression was significantly reduced by GSK-J4 treatment, albeit to a lesser extent than that in NIH3T3 cells (**Figure 1D** *vs* **1C**). The GSK-J4 inhibition of Shh target genes was further confirmed in the primary *Gli3^-/-^* MEF cells (**Figure 1E**). The basal *Gli1* mRNA level in *Gli3^-/-^* MEFs is higher than that in wildtype MEF since Gli3 is a repressor in the Shh signaling pathway. GSK-J4 treatment also significantly reduced *Gli1* basal expression levels in *Gli3^-/-^* MEFs. Given our finding that Jmjd3 plays an important role in the Shh signaling pathway (10), we examined whether GSK-J4 inhibition of Shh gene expression is indeed through targeting Jmjd3. When primary *Jmjd3^-/-^* MEFs were treated with or without GSK-J4 in the presence or absence of Shh, we observed that Jmjd3 deficiency largely blocked both the Shh-induced target gene expression and GSK-J4 inhibition (**Figures 1F, G**). To understand the molecular mechanism in which GSK-J4 inhibits the Shh signaling pathway, we performed an H3K27me3 ChIP assay. In NIH3T3 cells treated with GSK-J4, the H3K27me3 levels at the *Gli1* gene regulatory region near its promoter were significantly increased (**Figure 1H**), suggesting impaired demethylation activities of Jmjd3. Taken together, GSK-J4 blocked Shh signaling by inhibiting Jmjd3 demethylase activities.

### Growth of CGNP cells is sensitive to GSK-J4

**Figure 2:**
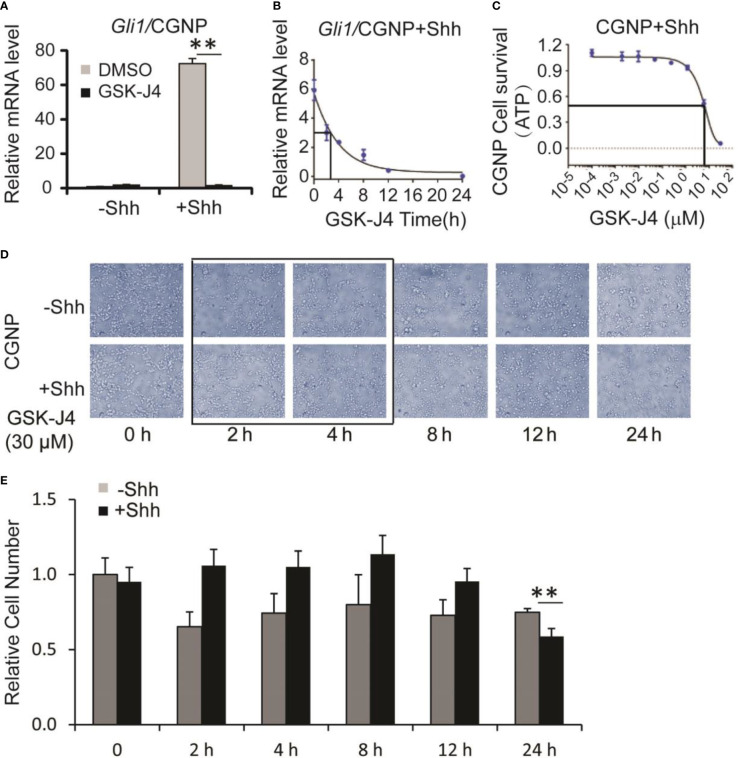
Shh signaling and growth of CGNP cells are inhibited by GSK-J4. **(A)** GSK-J4 decreases Shh-induced *Gli1* expression in P4 CGNP cells. **(B)** Time course of GSK-J4 effect on *Gli1* expression in P4 CGNP cells. **(C)** Dose dependent survival of CGNP cells after the treatment of GSK-J4 shown by ATP assay. **(D, E)** Cell performances after the treatment of GSK-J4 for indicated time courses and doses. The cells with frame have significant changes after treatment with GSK-J4. Significance determined using *t*-test. **p < 0.01.

Shh secreted from Purkinje neurons is essential for CGNP proliferation and cerebellar development (1). To determine whether GSK-J4 inhibits CGNP proliferation by inhibiting the Shh signaling pathway, we isolated primary CGNP cells from P4 mouse cerebella, and treated them with Shh conditioned media (37). Shh-induced target gene expression in CGNP cells was significantly impaired by 30 µM of GSK-J4 treatment for 24 hours (**Figure 2A**). To further investigate inhibition of GSK-J4 in detail, we treated CGNP cells with 30 µM of GSK-J4 for various durations. We observed that *Gli* mRNA levels were significantly decreased after 2 hours of treatment with GSK-J4, and the decrease reached 50% after 3 hours (**Figure 2B**). To quantitate the effects of GSK-J4 on CGNP cell growth, we treated the CGNP cells in the presence of Shh with various concentrations of GSK-J4 for 24 hours and measured ATP percentage to indicate cell survival. 6 µM of GSK-J4 treatment reduced 50% of cell growth in CGNP cells (**Figure 2C**). We further measured CGNP growth at various time points after 30 µM of GSK-J4 treatment. We observed no significant growth change until at 24 h time point (**Figure 2D** **, E**). Therefore, the decrease of *Gli1* expression after GSK-J4 treatment at earlier time points was not caused by GSK-J4 toxicity. These results confirmed that GSK-J4 inhibits the Shh signaling pathway in CGNP cells. Furthermore, at 24 h time point after GSK-J4 treatment, CGNP cell numbers were significantly reduced in Shh treated group, but not in the untreated one (**Figures 2D** **, E**), suggesting that the GSK-J4 inhibition of CGNP cell growth requires Shh signaling. Hence, consistent with our previous observation that Jmjd3 is required for Shh target gene expression and cerebellum development (10), pharmacological inhibition of Jmjd3 by GSK-J4 also efficiently inhibited Shh target gene expression during cerebellar development.

### GSK-J4 shows antitumor activity in medulloblastoma

**Figure 3:**
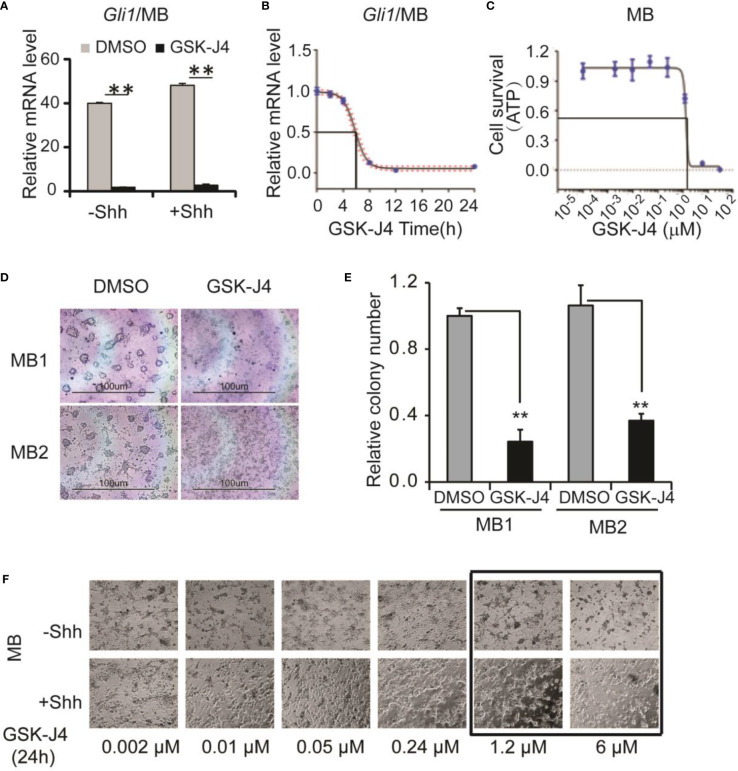
GSK-J4 shows antitumor activity in mouse primary medulloblastoma cells. **(A)** Shh target gene *Gli1* mRNA expression is inhibited by the treatment of GSK-J4 on mouse Shh-type primary medulloblastoma cells (MB). **(B)** Time course of GSK-J4 effects on *Gli1* expression in mouse primary medulloblastoma cells. **(C)** Dose dependent survival of primary medulloblastoma cells after the treatment of GSK-J4 shown by ATP assay. **(D)** Survival of primary medulloblastoma cells treated with GSK-J4. **(E)** Tumor colonies after treatment of GSK-J4. **(F)** Time course performances of primary medulloblastomas after the treatment of GSK-J4. The cells with frame have significant performance changes after treatment with GSK-J4. Significance determined using *t*-test. **p < 0.01.

Next, we determined whether GSK-J4 inhibits Shh-type medulloblastoma. We bred *Rosa26-SmoM2*, *actin-creER* transgenic mice, in which SmoM2 mutation causes constitutive Shh signaling and Shh-type medulloblastoma (9). From mouse medulloblastoma tumors, we cultured primary tumor cells and treated them with 30 µM of GSK-J4. *Gli1* expression was significantly inhibited as shown by RT-qPCR and the tumor cell growth was completely blocked by GSK-J4 as measured by ATP assay (**Figures 3A** **, C**), suggesting that GSK-J4 is more effective in inhibiting the growth of medulloblastoma cells compared to CGNP cells. To find the minimal treatment time of GSK-J4, we treated the tumor cells with 30 µM of GSK-J4 in a time course (**Figure 3B**) and found that the *Gli1* mRNA level decreased 50% after 6-7 hours. Consistently, most of the tumor cells were killed before 8 hours (**Figure 3B**). We next determined minimal concentration of GSK-J4 to inhibit medulloblastoma growth by treating the tumor cells with various concentrations of GSK-J4 for 24 hours. Our results suggested that 6 µM of GSK-J4 significantly inhibited the tumor cell growth (**Figures 3C–E**). Since Shh signaling is constitutively elevated in Shh-type medulloblastoma, Shh ligand had no significant effect on *Gli1* expression, and GSK-J4 showed similar effects on tumor cell growth in the presence or absence of Shh (**Figure 3F**).

### GSK-J4 inhibits the proliferation of medulloblastoma cells

**Figure 4:**
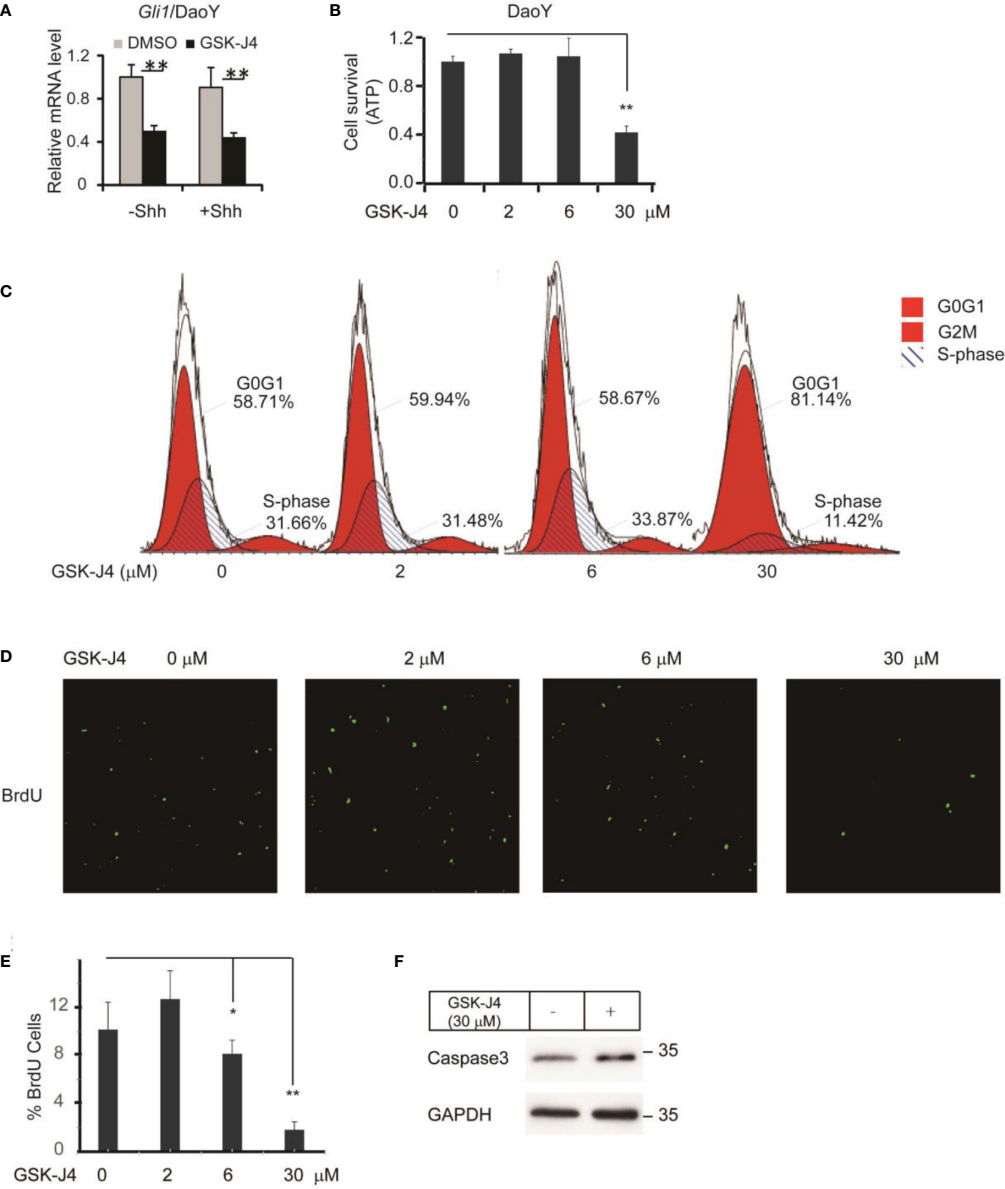
Growth of human DaoY medulloblastoma cells is inhibited and cells are arrested at G0/G1 by treatment of GSK-J4. **(A)** Shh target gene *Gli1* expression is impaired by GSK-J4 in human medulloblastoma cell line DaoY. **(B)** Growth of DaoY Cells is inhibited by GSK-J4. **(C)** Growth of DaoY Cells is arrested at G0/G1 phase with decrease of S-phase by GSK-J4. **(D, E)** The proliferation of DaoY cells is reduced by treatment of GSK-J4. **(F)** Apoptosis of DaoY tumor cells is not dramatically induced by GSK-J4. Significance determined using Student’s t-test; *p<0.05, **p<0.01.

To translate the finding that GSK-J4 is a candidate to treat medulloblastoma, we measured *Gli1* mRNA level changes before and after GSK-J4 treatments in a human Shh-type medulloblastoma cell line DaoY. *Gli1* was expressed actively in DaoY cells regardless of Shh treatment, and GSK-J4 significantly inhibited *Gli1* expression (**Figure 4A**). To determine the sensitivity of DaoY cells to GSK-J4 treatment, we measured cell survival under 2 µM, 6 µM or 30µM of GSK-J4 treatment, and found that 30 µM GSK-J4 significantly hindered cell viability of DaoY medulloblastoma cells (**Figure 4B**). To determine whether the cell number reduction was caused by inhibition of proliferation or increase of apoptosis, we performed cell cycle analyses. We found that 30µM of GSK-J4 treatment significantly induced cell cycle arrest at G0/G1 phase, with an extension at G0/G1phase and a reduction of S-phase (**Figure 4C**). BrdU incorporation assay in DaoY cell cultures further verified our hypothesis that GSK-J4 could inhibit DaoY medulloblastoma cell cycle progression. BrdU positive cells were significantly decreased upon 6 µM of GSK-J4 treatment, and 30 µM of GSK-J4 treatment showed further reduction (**Figures 4D** **, E**). After treatment with GSK-J4, Caspase3 levels were slightly increased as shown by western blot (**Figure 4F**), suggesting minor cell apoptosis caused by GSK-J4 treatment. Taken together, GSK-J4 inhibits the proliferation of human DaoY medulloblastoma cells.

### RNA-seq and bioinformatics analyses show biosynthesis of cholesterol as an additional target of GSK-J4

**Figure 5:**
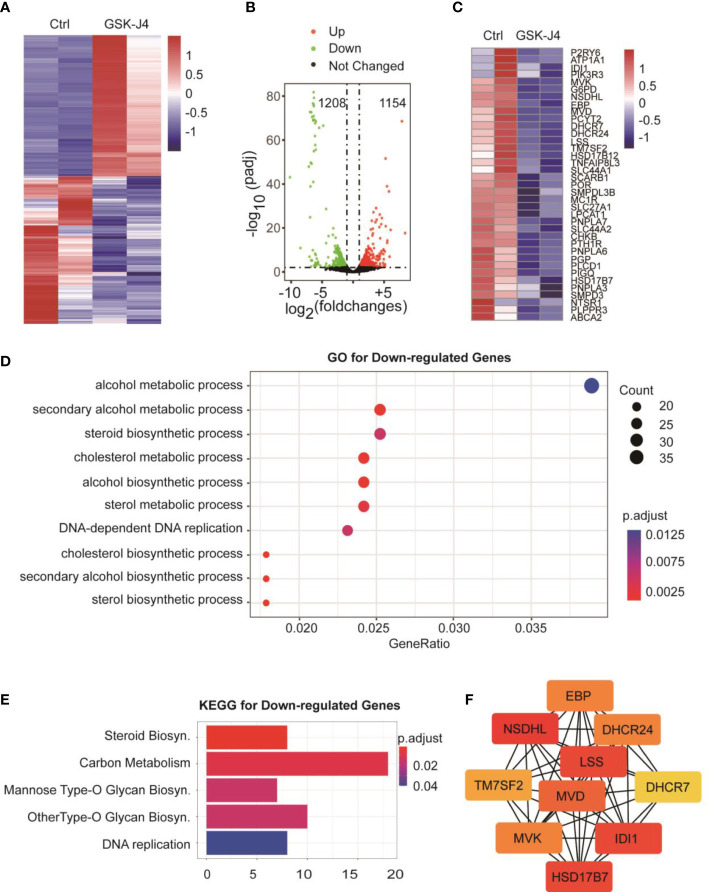
Biosynthesis of cholesterol and phospholipids are impaired by GSK-J4 in DaoY cells. **(A)** Heatmap of gene mRNA levels in DaoY cells after treatment with GSK-J4. **(B)** Volcano plot of the differentially expressed genes in DaoY cells after treatment with GSK-J4. **(C)** Heatmap of the mRNA level of cholesterol biosynthesis genes in DaoY cells after treatment with GSK-J4. **(D)** GO analysis of the down-regulated genes in DaoY cells treated with GSK-J4. **(E)** KEGG analysis of the down-regulated genes in DaoY cells treated with GSK-J4. **(F)** STRING analysis for key genes in cholesterol biosynthesis.

**Figure 6:**
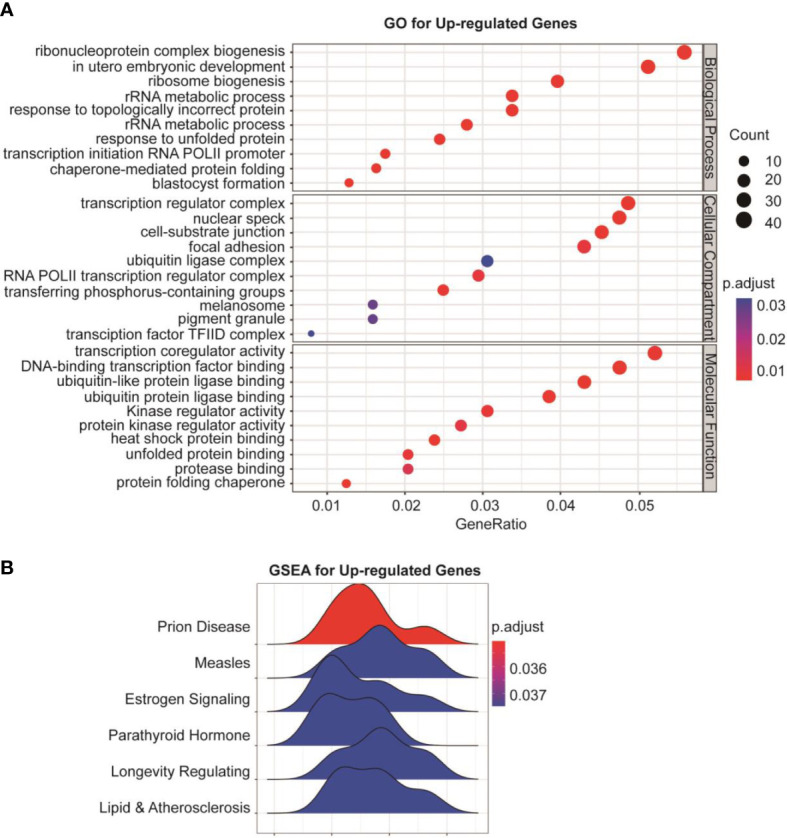
Protein degradation and unfolding are activated by GSK-J4 in DaoY cells. **(A)** GO analysis of the up-regulated genes in DaoY cells treated with GSK-J4. **(B)** GSEA analysis of the up-regulated genes in DaoY cells treated with GSK-J4.

To understand the global effects of GSK-J4 on medulloblastoma cells, we carried out RNA-seq of DaoY cells treated with or without GSK-J4 (n=2). Heatmap of mRNA levels showed comparable samples used for this assay (**Figure 5A**). Totally, 1208 genes had higher expression after treatment with GSK-J4, and 1154 genes decreased their expression (**Figure 5B**). The down-regulation of these genes possibly resulted from the inhibition of H3K27me3 demethylase activities, while the upregulation could be caused by indirect effects. GO analysis showed sterol biosynthesis genes were enriched in the down-regulated gene population (**Figure 5D**). The heatmap further confirmed the significant down-regulation of cholesterol biosynthesis genes (**Figure 5C**). Consistently, KEGG assay also showed steroid biosynthesis genes decreased after treatment with GSK-J4 (**Figure 5E**). Using STRING package, we identified the top 10 genes of sterol biosynthesis pathway as presented in **Figure 5F**, highlighting *MVD*, *DHCR7*, *DHCR24*, *LSS* and *HSD17B7* in cholesterol biosynthesis. On the other hand, the up-regulated genes mostly were associated with protein degradation and unfolding pathways (**Figure 6A**), which may result in dys-regulated metabolism and disease (**Figure 6B**).

## Discussion

We broadly tested an epigenetic inhibitor GSK-J4 in Shh-responsive tissue cultures, including Shh responsive cell models NIH3T3, primary MEF cells, primary CGNP cells and medulloblastoma cells. Importantly, we did pilot experiments in human DaoY Shh-medulloblastoma cell line. We provide strong evidences showing decreased mRNA levels of the key Shh target gene *Gli1* by GSK-J4 treatment in all settings. Since Gli1 is a positive feedback transcription factor in Shh signaling, it plays important roles to amplify Shh signals *in vivo*. One strategy for inhibiting Shh signaling in tumor growth is by inhibiting Gli1 levels (27).

Our results together showed that GSK-J4 is a potential inhibitor for Shh-type medulloblastoma growth (**Figures 4B–D**), and it can be developed for possible targeted therapy of pediatric Shh-type medulloblastoma. Hashizume et al. showed that GSK-J4 is a candidate for brainstem glioma therapy by inhibiting H3K27me3 demethylase (33). The group administered GSK-J4 by intraperitoneal injection, and observed active derivative GSK-J1 in brain tissues, suggesting the small molecule can reach cerebellum for medulloblastoma therapy. In our study, GSK-J4 treatment reduced active tumor cells and BrdU incorporation to genome. Cells were arrested at G0/G1 phase with significant decrease in S-phase after GSK-J4 treatment, together showing inhibited proliferation by GSK-J4.

**Figure 7:**
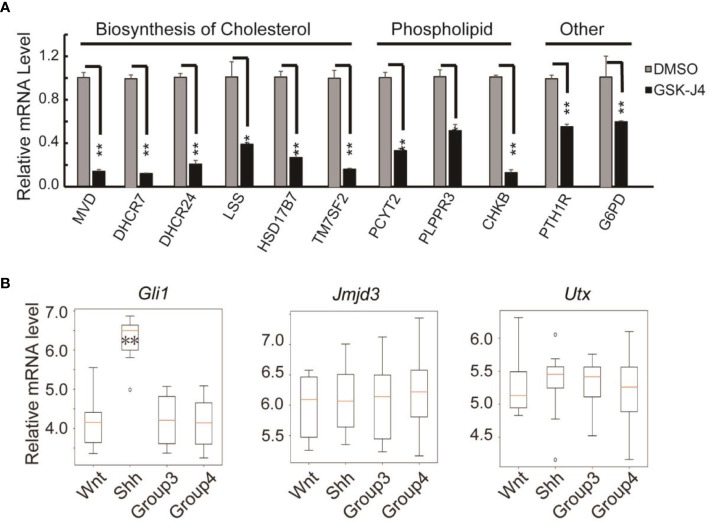
Verification of cholesterol and phospholipids biosynthesis gene expression decreases in DaoY cells treated with GSK-J4. **(A)** RT-qPCR verification of the expression of cholesterol and phospholipids biosynthesis genes in DaoY cells treated with GSK-J4. **(B)** Gene expression levels by microarray analysis in four subgroups of human medulloblastoma (GSE37418 (7)). Number of samples in each group, wnt: 8, Shh: 10, Group3: 17, Group4: 41. Significance determined using Student’s t-test. **p< 0.01, *p< 0.05.

Surprisingly, we found the expression of cholesterol biosynthesis genes was significantly reduced by GSK-J4 treatment. The key biosynthesis genes included *MVD*, *DHCR7*, *DHCR24*, *LSS*, *HSK17B7* and *TM7SF2* (**Figure 7A**), suggesting that the biosynthesis of cholesterol was impaired in cells treated with GSK-J4. These cholesterol biosynthesis genes were possibly directly regulated by Jmjd3. It was reported that the reduction of cholesterol biosynthesis resulted in S-phase decrease and G0/G1 extension (42), which is consistent with our current finding. Besides the essential role in cell physiological activities, cholesterol is a functional modification of the Shh signaling pathway proteins. Both Shh ligand and membrane protein Smoothed are covalently modified by cholesterol (43, 44). Hence, our study suggested GSK-J4 can also regulate Shh signaling by restraining cholesterol metabolism.

**Figure 8:**
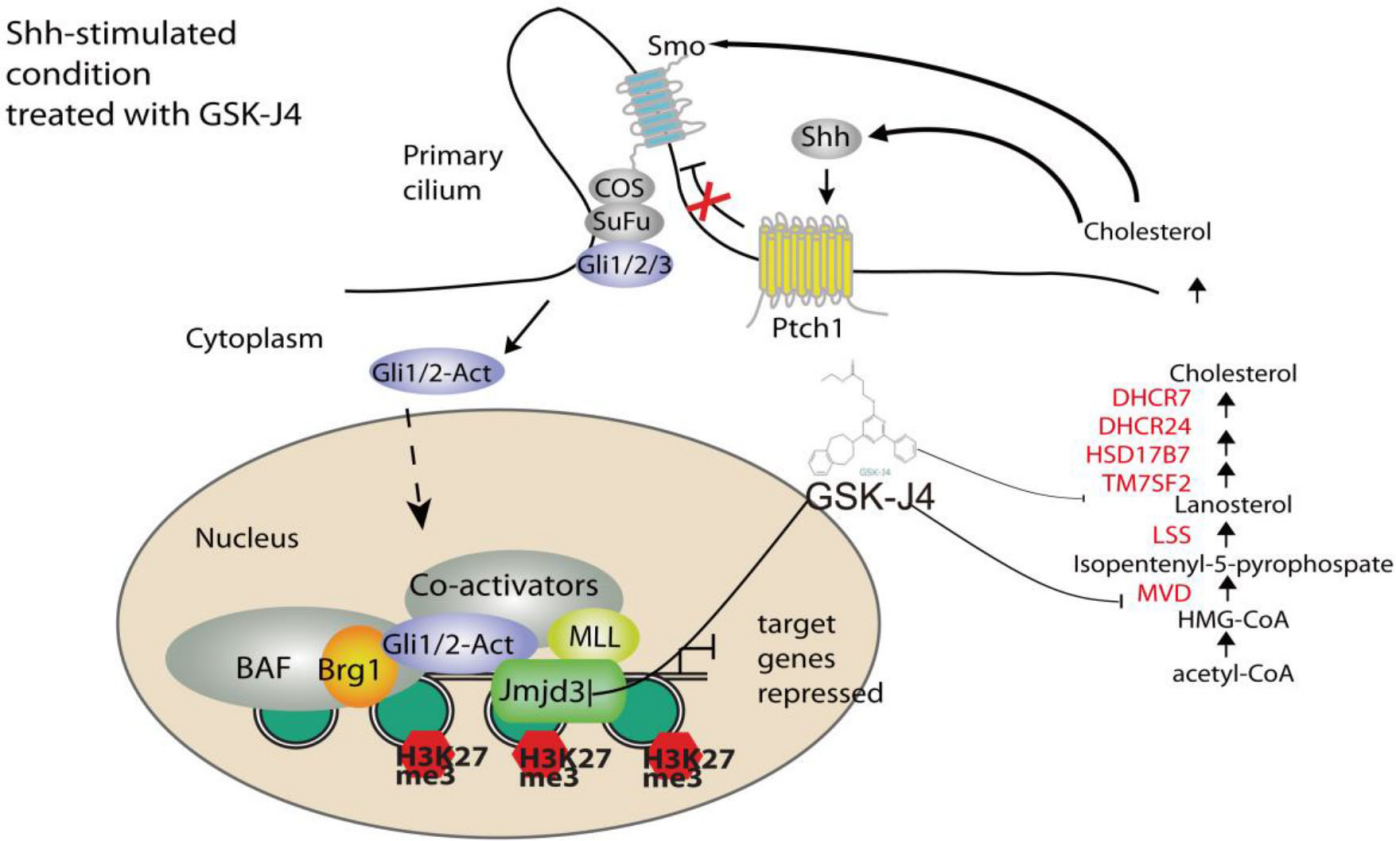
A model of Shh signaling regulated by small molecule compound GSK-J4 targeting the epigenetic switch. At basal condition, the processed transcriptional factor Gli3R represses Shh target gene and maintains high level of H3K27me3 on the regulatory region of target genes. In the presence of Shh, Gli3R is replaced with Gli1/2-Act, which recruits the coactivators Brg1 and Jmjd3. Decreases of H3K27me3 turn on the target gene expression. While acetyl-CoA is present, serial enzymes including MVD, DHCR7, and DHCR24, LSS convert acetyl-CoA to form cholesterol. With the treatment of GSK-J4, activity of Jmjd3 is inhibited and H3K27me3 increases, which leads to impaired Shh signaling and cholesterol biosynthesis.

GSK-J4 could inhibit the H3K27me3 demethylase activities of both Jmjd3 and Utx. We think the GSK-J4 effects on Shh signaling and medulloblastoma were largely through targeting Jmjd3. The two H3K27me3 demethylases have diverse functions in Shh-type medulloblastoma formation, though they have similar expression levels among different types of medulloblastoma (**Figure 7B**). Jmjd3 functions as a co-activator for Shh target genes, whereas UTX could inhibit medulloblastoma initiation by promoting tumor differentiation (29). Our study together with others’ showed that GSK-J4 is a promising agent for targeted therapy of human medulloblastoma, and other tumors dependent on Shh signaling and cholesterol metabolism (**Figure 8**). Hence, our study highlights the possibility of using GSK-J4 that targets H3K27me3 demethylase Jmjd3 to treat Shh medulloblastoma patients.

## Data availability statement

The datasets presented in this study can be found in online repositories. The names of the repository/repositories and accession number(s) can be found in the article/supplementary material.

## Ethics statement

The animal study was reviewed and approved by University of Texas Southwestern Medical Center.

## Author contributions

JW and XS designed the experiments. HD, XG, XS, NF, YL and BL performed the experiments, collected the data and analysed the results. XS, SL, and JW wrote the paper. All authors contributed to the article and approved the submitted version.

## Funding

This study was supported by Grants for Scientific Research of BSKY (XJ2020039) from Anhui Medical University, the Natural Science Foundation of China (82073124), and Scientific Research Platform and Base Upgrading Plan of Anhui Medical University (2021xkjT048).

## Acknowledgments

We appreciate the *Jmjd3^-/-^* MEF cells from Dr. Akira from Japan.

## Conflict of interest

The authors declare that the research was conducted in the absence of any commercial or financial relationships that could be construed as a potential conflict of interest.

## Publisher’s note

All claims expressed in this article are solely those of the authors and do not necessarily represent those of their affiliated organizations, or those of the publisher, the editors and the reviewers. Any product that may be evaluated in this article, or claim that may be made by its manufacturer, is not guaranteed or endorsed by the publisher.
