# Supplementary material for: Targeting H3K27me3 demethylase to inhibit Shh signaling and cholesterol metabolism in medulloblastoma growth
Source: Front Oncol. 2022 Dec 1;12:1057147. doi: 10.3389/fonc.2022.1057147 (PMC9753684; doi:10.3389/fonc.2022.1057147)
Supplement: Supplementary file 1 [file Table_1.docx]

| RT-PCR primers | Sequence |
| --- | --- |
| Gli1_F | TCCGCGCCTCTCCCACATAC |
| Gli1_R | AGGGGTCGGAGACAGCATGG |
| Gapdh_F | GTGGTGAAGCAGGCATCTGA |
| Gapdh_R | GCCATGTAGGCCATGAGGTC |
| GLI1_F | GATGACCCCACCACCAATCA |
| GLI1_R | GAAAAGAGTGGGCCCTCGG |
| CHKB_F | AAGCAAGGCCCACAGACTAC |
| CHKB_R | ATGCCAGAGCATACCGACTG |
| DHCR7_F | CAGGACTTTAGCCGGTTGAGA |
| DHCR7_R | CAGCCATTGGGCCCTCC |
| PTH1R_F | GAAGCTGCTCAAATCCACGC |
| PTH1R_R | CTCAGCTTGTACCTCGCCAT |
| TM7SF2_F | ACACATGACGGGTTTGGCTT |
| TM7SF2_R | GGAAGATGTAGTAACCAGTAGCATT |
| DHCR24_F | GACCTCCATTGGCTGGACTC |
| DHCR24_R | GGTCTGAGTTTTCGGACGGA |
| LSS_F | CGGAGGGCACGTGTCTG |
| LSS_R | ATTCTTGGTGTCCAGCCCC |
| PLPPR3_F | GGAGGAGACGCGCTTTGTG |
| PLPPR3_R | ACTATGGGCAGCTCCACGA |
| HSD17B7_F | TTGGCCATTTTATCCTGATTCGG |
| HSD17B7_R | GCTGTAGGGTTCCTTGCCTT |
| G6PD_F | ACGACGAAGCGCAGACAG |
| G6PD_R | TCTTCTTCTTGGCCAGGTCAC |
| PCYT2_F | GGTGCGATGGCTGCTATGA |
| PCYT2_R | CACCTCGTCCACCCATTTGA |
| MVD_F | GTCATCAAGTACTGGGGCAAG |
| MVD_R | TTCAGCCAAATCCGGTCCTC |
| GAPDH_F | TCTCGAGGTTCGCTGCTTTT |
| GAPDH_R | TCGGAGTCAACGGATTTGGT |
|  |  |

| ChIP-PCR primers | Sequence |
| --- | --- |
| Gli1_F | CGCTCACTTCCCTCGTATATCCTTC |
| Gli1_R | GGCAGTATAGGGTCCCTCAAGGG |
|  |  |
